# Thymidylate Synthase (TYMS) and Methylenetetrahydrofolate Reductase (MTHFR) Gene Polymorphisms Associated With Severe Capecitabine Toxicity: The First Case From Saudi Arabia

**DOI:** 10.7759/cureus.49215

**Published:** 2023-11-22

**Authors:** Nedal Bukhari, Hani Al-Mohanna, Fahad Almsned

**Affiliations:** 1 Department of Medical Oncology, King Fahad Specialist Hospital, Dammam, SAU; 2 Department of Internal Medicine, Imam Abdulrahman Bin Faisal University, Dammam, SAU; 3 Department of Medical Oncology, Prince Sultan Military Medical City, Riyadh, SAU; 4 Department of Epidemiology and Public Health, King Fahad Specialist Hospital, Dammam, SAU; 5 Department of Population Health Management, Eastern Health Cluster, Dammam, SAU; 6 Department of Research and Development, Novo Genomics, Riyadh, SAU

**Keywords:** colon cancer, capecitabine, 5-fluorouracil, thymidylate synthase, methylenetetrahydrofolate reductase, dihydropyrimidine dehydrogenase

## Abstract

Dihydropyrimidine dehydrogenase (DPD) is the major enzyme in the catabolism of fluoropyrimidine chemotherapy. Deficiencies in this enzyme level typically predispose patients to fluoropyrimidine toxicities, and they are often linked to *DPYD* gene polymorphisms. Other gene polymorphisms such as thymidylate synthase (*TYMS*) and methylenetetrahydrofolate reductase (*MTHFR*) may induce similar toxicities. We report a patient with resected stage III colon cancer presenting with severe toxicity to adjuvant capecitabine, a prodrug of 5-fluorouracil (5-FU). Her *DPYD* gene sequencing was normal. However, the patient was heterozygous for c.1298A>C (p.E429A) in the methylenetetrahydrofolate reductase (*MTHFR*) gene and c.*450_*455del in the thymidylate synthase (*TYMS*) gene. The capecitabine dose was reduced in subsequent treatments and then titrated up gradually with no major side effects reported.

## Introduction

Capecitabine, a prodrug of 5-fluorouracil (5-FU), has been combined with oxaliplatin in the adjuvant treatment of colon cancer [1,2]. Most people tolerate capecitabine and 5-FU treatments [3]. However, a small percentage of patients may experience severe toxicity with those agents, which may be attributed to the deficiency of enzymes such as dihydropyrimidine dehydrogenase (DPD), methylenetetrahydrofolate reductase (MTHFR), and thymidylate synthase (TYMS) [4]. We herein report a patient with severe toxicity to the standard dose of adjuvant capecitabine secondary to *TYMS* and *MTHFR* gene polymorphisms.

## Case presentation

A 54-year-old female with a history of abdominal pain and episodic lower gastrointestinal bleeding initially presented to a local hospital, where she was found to be anemic; further investigations showed a malignant mass involving her sigmoid colon. The patient underwent a sigmoidectomy shortly afterward, which was remarkable for a T3N1 moderately differentiated adenocarcinoma. One out of five lymph nodes examined showed metastasis. The surgical margins were clear, and the patient was diagnosed with stage III colon cancer. She was scheduled for eight cycles of adjuvant capecitabine and oxaliplatin (XELOX), a 21-day cycle chemotherapy regimen.

The patient received her first cycle of full standard dose adjuvant XELOX four weeks after her surgery at her local hospital. The dosage was 130 mg/m^2^ of oxaliplatin on day 1 and 1250 mg/m^2^ of capecitabine twice daily (BID) for 14 days. Closer to the end of her capecitabine course, she developed mouth sores and diarrhea; both worsened after completing capecitabine. The patient presented to the emergency department of the same hospital immediately after completing cycle number 1 of chemotherapy with intermittent abdominal pain, grade III mucositis, and grade III diarrhea complicated by clinical dehydration. She was also found to be pancytopenic and thought to be septic. Her clinical condition prompted an admission, where she was started on broad-spectrum antibiotics and intravenous rehydration. Her symptoms markedly improved throughout her stay, which lasted almost 10 days. She had loose bowel motions on discharge, for which she used loperamide.

The patient sought medical advice and further treatment at our center. She was 14 days overdue for cycle number 2, and by the time she presented to our clinic, her diarrhea had stopped, and her mucositis had healed. Her complete blood count (CBC) parameters are listed in Table 1. After the first encounter, the patient's blood sample was sent for *DPYD*, *MTHFR*, and *TYMS* gene sequencing. Moreover, her adjuvant XELOX was reduced to a 50% dose. The plan also comprised further escalation of capecitabine by 5%-10% based on the patient's tolerance. The standard capecitabine dosage in the XELOX regimen at our institute is 1000 mg/m^2^ BID. The patient tolerated cycle number 2 at 500 mg/m^2^ BID without significant toxicity, except for a transient grade I peripheral neuropathy involving the tips of her fingers and toes bilaterally, which was believed to be oxaliplatin-related. A further increase in her capecitabine by 10% was performed at cycle number 3. Based on the gene sequencing results, the patient was found to be heterozygous for an indel *TYMS* variant (NM_001071.4(TYMS):c.*450_*455del, database for single nucleotide polymorphism {dbSNP}: rs11280056) and heterozygous for a missense *MTHFR* variant (c.1298A>C (p.E429A)), which are indicated in Table 2. No clinically relevant variants were reported for *DPYD*.

**Table 1:** CBC results CBC, complete blood count; Hb, hemoglobin

CBC	Results	Reference
Hb	9.5 g/dL	12-16 g/dL
WBC	7 × 10^9^/L	4-11 × 10^9^/L
Neutrophils	2.9 × 10^9^/L	1.5-7.5 × 10^9^/L
Platelets	170 × 10^9^/L	150-450 × 10^9^/L

**Table 2:** Whole-gene sequencing results of DPYD, TYMS, and MTHFR genes TYMS, thymidylate synthase; MTHFR, methylenetetrahydrofolate reductase

Gene	Results
DPYD	Normal
TYMS	Heterozygous variant of c.*450_*455del
MTHFR	Heterozygous variant of c.1298A>C (p.E429A)

We continued to escalate her capecitabine dose by 5%-10%; the maximal safe dose in her case was at 1000 mg/m^2^ BID, reached at cycle number 7 and continued through cycle number 8. Her peripheral neuropathy progressed, interfering with her daily activities, reaching a grade III level; oxaliplatin was discontinued after cycle number 6. After completing her chemotherapy, the patient was placed on surveillance, including a six-monthly CT of the chest, abdomen, and pelvis; carcinoembryonic antigen (CEA) level tests; and regular follow-ups. She is over three years out of adjuvant treatment and continues to do well with no residual neuropathy.

## Discussion

DPD, TYMS, and MTHFR are essential enzymes for the metabolism of 5-fluoropyrimidine-based chemotherapy [5,6]. DPD metabolizes over 80% of 5-FU in the liver. The conversion of 5-FU to inactive dihydrofluorouracil (DHFU) occurs through the DPD enzyme [6-8]. TYMS is the enzyme that converts deoxyuridine monophosphate (dUMP) to deoxythymidine monophosphate (dTMP), which is critical for DNA synthesis. The active metabolite of 5-FU forms a complex with thymidylate synthase (TYMS) and 5,10-methylenetetrahydrofolate (5,10-MTHF) and blocks dTMP production, leading to reduced DNA synthesis, and dUMP misincorporation into the DNA, resulting in DNA breakdown [9,10].

MTHFR plays an important role in the metabolism of folic acid. The substrate for MTHFR, 5,10-MTHF, is essential for converting dUMP to dTMP, whereas the product of 5-methyltetrahydrofolate is the methyl donor for the synthesis of adenosylmethionine and methionine in methylation reactions [9,10].

This case is heterozygous for a 5′ untranslated region (UTR) indel *TYMS* variant (NM_001071.4(TYMS):c.*450_*455del, dbSNP: rs11280056) [11]. This variant is known to affect TYMS activity levels. The TYMS expression is associated with sensitivity to 5-FU, and the TYMS enzyme is one of the 5-FU chemotherapy targets. While this functional link to fluoropyrimidine metabolism and tumor response has been demonstrated in multiple studies, the impact of genetic polymorphisms in *TYMS* is less clear [4,5]. The TYMS alleles have been reported in a few studies as associated with increased toxicity and sensitivity to fluoropyrimidines [11,12].

The rs45445694 polymorphism is the defining variant of the TYMS "2R" allele, associated with severe toxicities, in either heterozygosity or homozygosity. This allele is in the 5′ UTR and is a duplication of a 28-base pair repeat. This same locus can have variable tandem repeats between zero and nine copies, and studies suggest that increased copy numbers of the repeat are associated with an increase in TYMS expression [12,13]. An additional variant in *TYMS* has been found associated with toxicities to fluoropyrimidines: a 3′ UTR nine-base pair insertion/deletion polymorphism (rs11280056) [5,11-13].

Sibani et al. stated previously that there are 29 rare mutations in the *MTHFR* gene that are identified as homocystinuric, whereas two common variants, Ala222 to Val (A222V; 607093.0003) and Glu429 to Ala (E429A; 607093.0004), exist in patients with mild enzymatic activity [12].

It has been hypothesized that a decrease in MTHFR activity could lead to an increase in the concentration of 5,10-MTHF and, subsequently, the cytotoxicity to fluoropyrimidines. The MTHFR enzyme converts 5,10-MTHF to 5-methyltetrahydrofolate, which is important in methionine synthesis, nucleotide synthesis, and the DNA methylation process [12,13].

The *MTHFR* gene is located on chromosome 1p36.3, with several single-nucleotide polymorphisms (SNPs) reported. Two common SNPs are associated with decreased enzyme activity in homozygous patients: the MTHFR 1298A>C polymorphism induces a Glu to Ala substitution in a regulatory environment (30%-40% reduction), and the MTHFR 677C>T polymorphism is associated with an Ala to Val substitution in the catalytic domain (70% reduction in activity) [13,14]. Heterozygous individuals have a 40%-50% decrease in enzyme levels [13,14].

In this case, we applied the principles of genotype-based dosing of 5-FU and capecitabine used in *DPYD* gene polymorphisms with no further toxicity [8]. We concluded that a 1000 mg/m^2^ twice daily dose of capecitabine is safe in the presence of the *TYMS* and *MTHFR* polymorphism found in this patient. Although polymorphisms in *MTHFR* and *TYMS* genes are extremely rare causes of fluoropyrimidine toxicity, our findings support that *TYMS* c.*450_*455del (dbSNP: rs11280056) and the *MTHFR* c.1298A>C (p.E429A) variants are associated with toxicities to capecitabine in this case. Gene sequencing can give further clarification and answers to patients. Furthermore, it can improve the understanding of the disease mechanisms associated with capecitabine- and 5-FU-related toxicities.

## Conclusions

We present a Saudi female with clinical manifestations of DPD enzyme deficiency when exposed to 1250 mg/m^2^ BID dosing of capecitabine due to *TYMS* c.*450_*455del (dbSNP: rs11280056) and *MTHFR* c.1298A>C (p.E429A) gene variants. This case report demonstrates that *TYMS* and *MTHFR* gene polymorphisms are well-recognized causes of capecitabine toxicity. Additionally, it implies that gene sequencing aids in both individualizing treatment and forecasting harm associated with fluoropyrimidines. To our knowledge, this is the first case reported from our region.
